# Supplementary material for: Premature Macrophage Activation by Stored Red Blood Cell Transfusion Halts Liver Regeneration Post-Partial Hepatectomy in Rats
Source: Cells. 2022 Nov 7;11(21):3522. doi: 10.3390/cells11213522 (PMC9654889; doi:10.3390/cells11213522)
Supplement: Supplementary file 1 [file cells-11-03522-s001.zip › cells-1929637-supplementary.pdf]

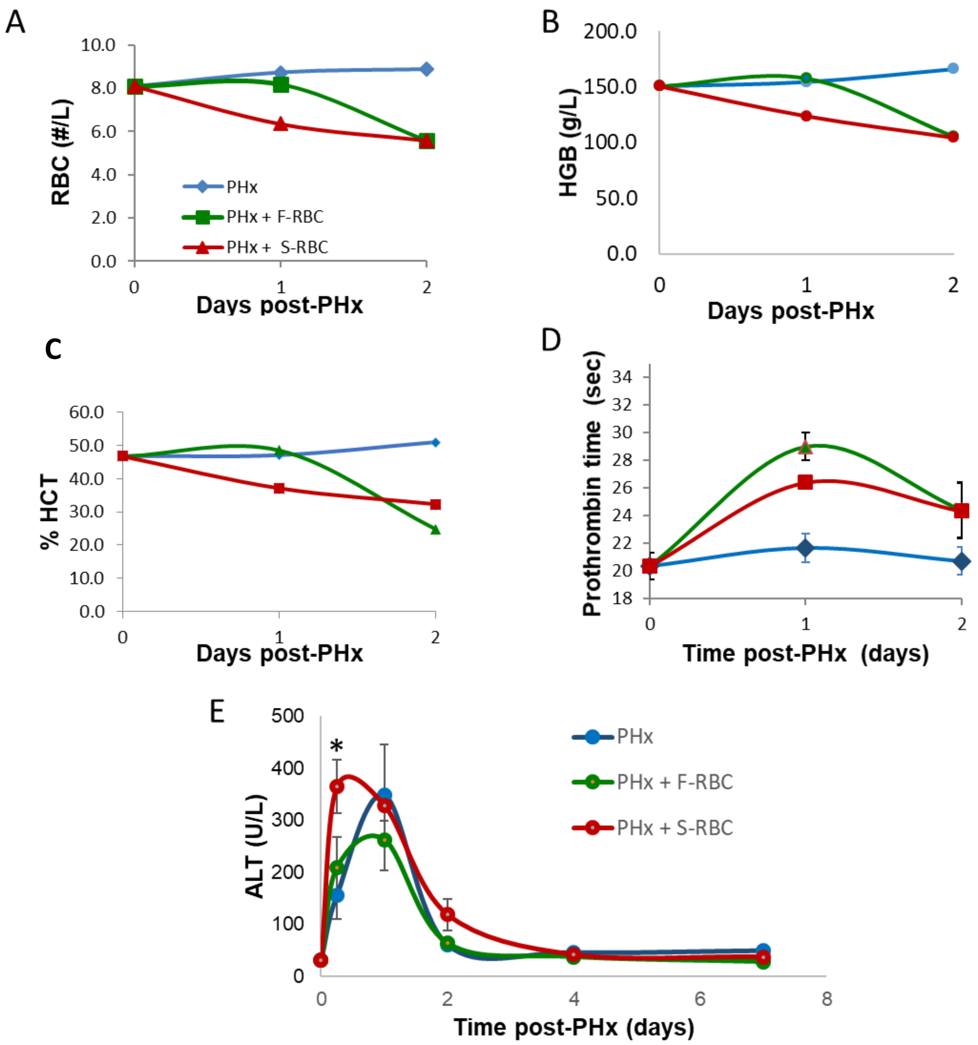

Figure S1. (**A**) RBC count, (**B**) hemoglobin levels, and (**C**) hematocrit one day post-PHx in the experimental groups. (**D**) Prothrombin time (**E**) ALT

Pathway	pValue	qValue	SetSize	PathSize
Adherens junction	1.17E-05	1.61E-03	65	74
Regulation of actin cytoskeleton	1.59E-05	1.61E-03	202	221
Cell cycle	2.51E-05	1.61E-03	121	127
Focal adhesion	4.23E-05	1.61E-03	185	206
Platelet activation	4.45E-05	1.61E-03	112	127
Endocytosis	4.55E-05	1.61E-03	260	288
Gap junction	4.75E-05	1.61E-03	79	88
MicroRNAs in cancer	5.44E-05	1.64E-03	133	143
Lysosome	6.75E-05	1.83E-03	119	129
Epstein-Barr virus infection	1.04E-04	2.36E-03	193	229
DNA replication	1.16E-04	2.42E-03	32	36
Thyroid hormone signaling pathway	1.45E-04	2.54E-03	112	119
Hippo signaling pathway	1.50E-04	2.54E-03	146	156
Leukocyte transendothelial migration	2.79E-04	3.97E-03	111	120
Proteoglycans in cancer	3.37E-04	4.56E-03	190	203
2-Oxocarboxylic acid metabolism	3.77E-04	4.82E-03	18	19
Hepatitis B	5.46E-04	6.43E-03	122	139
ECM-receptor interaction	6.19E-04	6.67E-03	75	84
Chemokine signaling pathway	6.32E-04	6.67E-03	170	178
Tight junction	6.40E-04	6.67E-03	127	143
Sphingolipid signaling pathway	8.08E-04	7.82E-03	110	124
cGMP-PKG signaling pathway	1.01E-03	9.43E-03	157	171

Supplemental Table S1: Molecular pathways that were altered between rats transfused with F-RBCs and those receiving S-RBCs
